# Endoscopic ultrasound localisation of completely embedded oesophageal fish bone and endoscopic retrieval

**DOI:** 10.1055/a-2839-9668

**Published:** 2026-04-20

**Authors:** Chaonan Chen, Jin Liu, Xinyu Fan, Junmei Jiang

**Affiliations:** 1 34708 Department of Gastroenterology, Shandong Provincial Hospital Affiliated to Shandong First Medical University, Jinan, China

Foreign body ingestion and food bolus impaction are common in clinical practice 1 . Esophageal perforation is a serious and potentially life-threatening complication, particularly with sharp objects such as fish bones 2 3 . Intramural esophageal foreign bodies are rare and may be missed on conventional endoscopy, resulting in delayed diagnosis and increased complications. This video highlights the diagnostic value of endoscopic ultrasonography (EUS) and demonstrates a safe, minimally invasive treatment strategy ( Video 1 ).

Endoscopic ultrasound localisation of the completely embedded oesophageal fish bone and endoscopic retrieval.Video 1

**Fig. 1:**
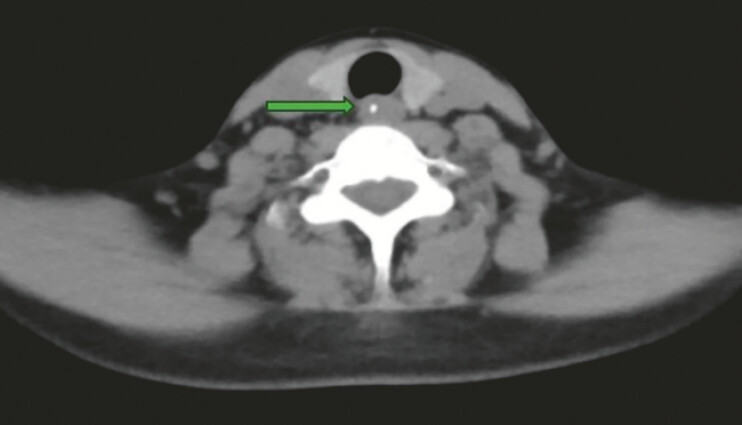
Cervical computed tomography showing a linear hyperdense lesion (~2.3 cm) in the upper esophagus at the C6–C7 level, with esophageal wall thickening and luminal narrowing, suggestive of an esophageal foreign body.

A 39-year-old woman presented with odynophagia lasting 3 days after eating fish. Cervical computed tomography revealed a linear hyperdense lesion (~2.3 cm) in the upper esophagus at the C6–C7 level, with wall thickening and luminal narrowing, suggesting a foreign body ( Fig. 1 ).

Conventional esophagogastroduodenoscopy localized the lesion approximately 18 cm from the incisors and showed mucosal hyperemia, edema, and focal disruption; however, no foreign body was detected. A smooth submucosal bulge with an intact surface was observed opposite to the suspected penetration site.

**Fig. 2:**
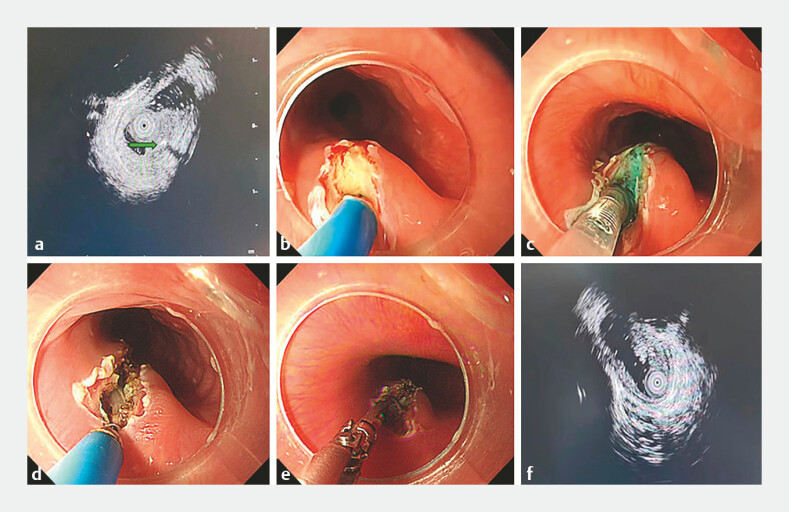
**a** Endoscopic ultrasonography showing a hypoechoic submucosal lesion with a linear hyperechoic structure and posterior acoustic shadowing (green arrow), consistent with an intramural esophageal foreign body. **b** Longitudinal mucosal incision over the submucosal bulge using an endoscopic incision knife, with drainage of the purulent material. **c** Submucosal injection of a mixed solution achieving adequate lifting. **d** Stepwise endoscopic dissection exposing the embedded fish bone. **e** Successful removal of the fish bone using biopsy forceps. **f** Follow-up endoscopic ultrasonography showing no residual foreign body.

EUS demonstrated a hypoechoic submucosal lesion containing a linear hyperechoic structure with posterior acoustic shadowing, consistent with an intramural esophageal foreign body ( Fig. 2 **a** ). Combined imaging and endoscopic findings localized the foreign body to the submucosal bulge rather than the mucosal injury, confirming a fish bone completely embedded in the esophageal wall.

**Fig. 3:**
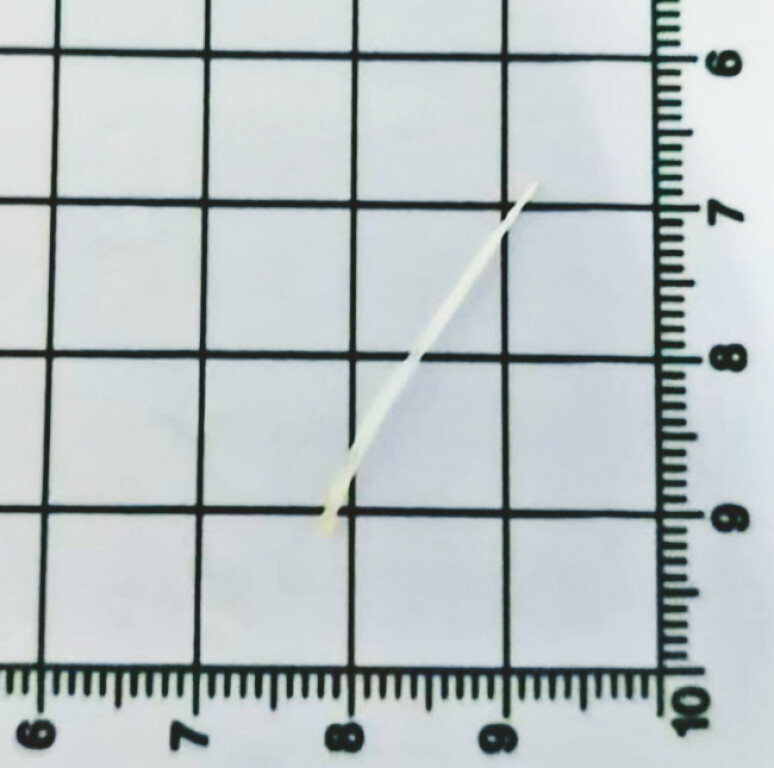
The removed fish bone measured approximately 2.7 cm.

A longitudinal mucosal incision over the bulge was performed using an endoscopic incision knife, resulting in purulent drainage ( Fig. 2 **b** ). After submucosal injection ( Fig. 2 **c** ), stepwise dissection exposed the embedded fish bone, which was removed with biopsy forceps ( Fig. 2 **d–e** ). Follow-up EUS confirmed complete removal with an intact muscularis propria ( Fig. 2 **f** ). The fish bone measured approximately 2.7 cm ( Fig. 3 ). The defect was left open to allow adequate drainage, and clip placement at this proximal esophageal location could have caused swallowing discomfort.

The mucosal ulcer was allowed to heal spontaneously. After thorough endoscopic irrigation, the patient was nil per os for 3 days with antibiotic prophylaxis, without chest pain, fever or other discomfort. The diet was then switched to liquid, and the patient recovered well and was discharged.

In conclusion, when an esophageal foreign body is suspected despite negative endoscopy, EUS enables accurate localization and safe endoscopic removal, avoiding surgery.

Endoscopy_UCTN_Code_TTT_1AO_2AL Endoscopy_UCTN_Code_CCL_1AB_2AF Endoscopy_UCTN_Code_TTT_1AS_2AB
